# A Novel Phonology- and Radical-Coded Chinese Sign Language Recognition Framework Using Accelerometer and Surface Electromyography Sensors

**DOI:** 10.3390/s150923303

**Published:** 2015-09-15

**Authors:** Juan Cheng, Xun Chen, Aiping Liu, Hu Peng

**Affiliations:** 1 Department of Biomedical Engineering, Hefei University of Technology, 193 Tunxi Road, Hefei 230009, China; E-Mails: chengjuan@hfut.edu.cn (J.C.); xun.chen@hfut.edu.cn (X.C.); hpeng@hfut.edu.cn (H.P.); 2 Department of Electrical and Computer Engineering, University of British Columbia, Vancouver, BC V6T-1Z4, Canada; E-Mail: aipingl@ece.ubc.ca

**Keywords:** sign language recognition, component level classification, accelerometer, electromyography, hidden markov model (HMM), dynamic time warping

## Abstract

Sign language recognition (SLR) is an important communication tool between the deaf and the external world. It is highly necessary to develop a worldwide continuous and large-vocabulary-scale SLR system for practical usage. In this paper, we propose a novel phonology- and radical-coded Chinese SLR framework to demonstrate the feasibility of continuous SLR using accelerometer (ACC) and surface electromyography (sEMG) sensors. The continuous Chinese characters, consisting of coded sign gestures, are first segmented into active segments using EMG signals by means of moving average algorithm. Then, features of each component are extracted from both ACC and sEMG signals of active segments (*i.e.*, palm orientation represented by the mean and variance of ACC signals, hand movement represented by the fixed-point ACC sequence, and hand shape represented by both the mean absolute value (MAV) and autoregressive model coefficients (ARs)). Afterwards, palm orientation is first classified, distinguishing “Palm Downward” sign gestures from “Palm Inward” ones. Only the “Palm Inward” gestures are sent for further hand movement and hand shape recognition by dynamic time warping (DTW) algorithm and hidden Markov models (HMM) respectively. Finally, component recognition results are integrated to identify one certain coded gesture. Experimental results demonstrate that the proposed SLR framework with a vocabulary scale of 223 characters can achieve an averaged recognition accuracy of 96.01% ± 0.83% for coded gesture recognition tasks and 92.73% ± 1.47% for character recognition tasks. Besides, it demonstrats that sEMG signals are rather consistent for a given hand shape independent of hand movements. Hence, the number of training samples will not be significantly increased when the vocabulary scale increases, since not only the number of the completely new proposed coded gestures is constant and limited, but also the transition movement which connects successive signs needs no training samples to model even though the same coded gesture performed in different characters. This work opens up a possible new way to realize a practical Chinese SLR system.

## 1. Introduction

Sign language (SL) is the predominant approach for deaf community communication, and it is often regarded as the most structured among the various gesture categories of a symbolic nature. The aim of sign language recognition (SLR) is to provide not only an efficient and accessible pathway for the communication between the deaf and the hearing, but also a convenient and natural input form for human computer interaction interface [1,2,3]. In recent years, a number of sign languages worldwide have been studied for recognition purpose, including American Sign Language (ASL) [4,5,6,7,8,9,10], Arabic Sign Language [11,12,13], Greek Sign Language [14,15,16], Korean Sign Language [17,18,19] and Chinese Sign Language (CSL) [20,21,22] *etc*.

**Figure 1:**
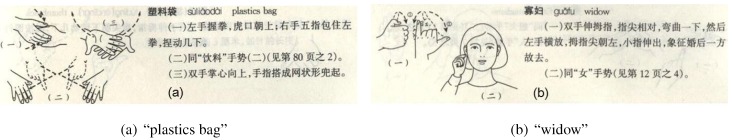
Sign gestures edited by China Association of the Deaf. (**a**) Chinese word “plastics bag”; (**b**) Chinese word “widow”.

**Figure 2:**
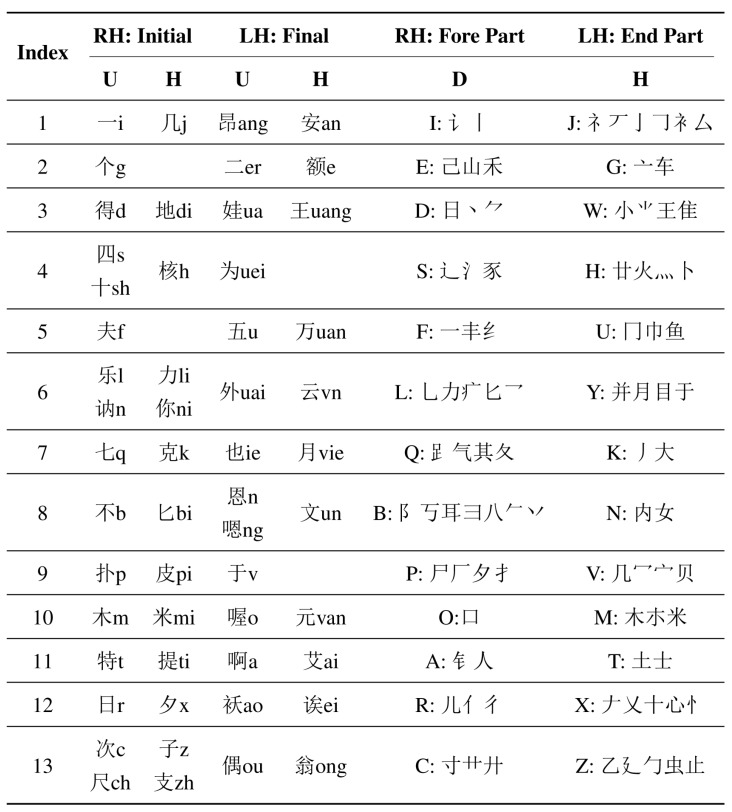
Typical hand shapes and finger orientations. (RH: right hand; LH: left hand; U: upward; H: horizontal; D: download.)

**Figure 3:**
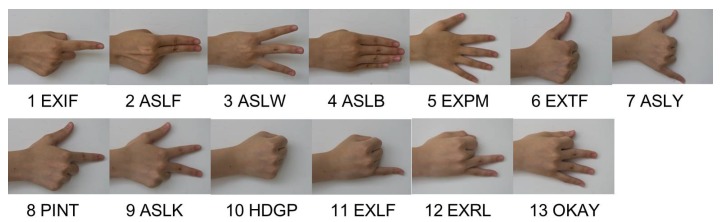
The basic hand shapes with index from 1 to 13.

**Figure 4:**
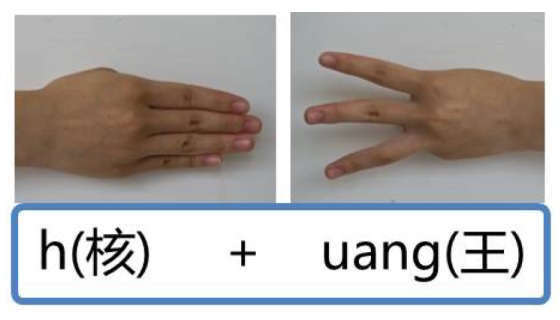
The phonology-coded gestures of Chinese character pronunciation “huang”.

**Figure 5:**
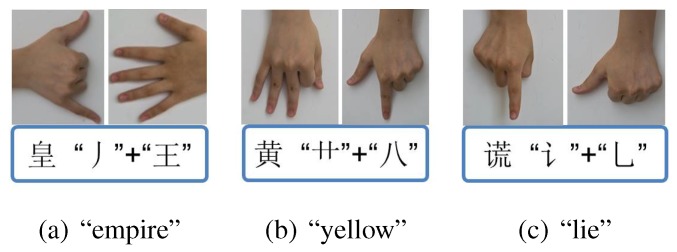
Component-coded gestures of characters sharing the same pronunciation “huang”. (**a**) Chinese character “empire”; (**b**) Chinese character “yellow”; (**c**) Chinese character “lie”.

It is reported that there are 20.57 million deaf people living in China, accounting for about a quarter in the world [23]. Implementing an automatic CSL recognition system is highly necessary for the disabled. Currently, the sign gestures employed by CSL recognition systems are generally referred to the manual edited by China Association of the Deaf [24]. The number of the normalized sign gestures is over 5000. However, the number of frequently used Chinese characters is about 3500 and the number of frequently used words is far more than tens of thousands. It is obvious that the normalized sign gestures referred to the manual is not enough for practical usage. Besides, most of these sign gestures, similar to other international SLs, are symbolic and consist of complex components. For instance, Figure 1 displays two sign gestures of the manual (the signing of “plastics bag” is shown in Figure 1a and that of “widow” is shown in Figure 1b). Both of them contain more than two kinds of hand shapes and two kinds of hand movements, which sometimes are hard to be standardized due to regional differences and individual comprehension styles. Meanwhile, it is found that sign gestures referred to the manual sometimes share the same subword. For instance, the sign gesture of “drinks” is included in the sign gestures of “plastics bag”, and the sign gesture of “woman” is included in the sign gestures of “widow”. Additionally, the same hand shape (*i.e.*, palm extension) but with different hand orientation (upward and downward) are included in the sign gestures of “plastics bag”. Consequently, a wide variety of solutions have been proposed for possible performance improvement, such as more advanced classifiers and component-level recognition framework [6,21,25,26,27,28]. However, due to the fact that the sign gesture is complex and variant, the number of movement components cannot be constant or maintain at a low level when the number of the vocabulary increases. Designing continuous and large-vocabulary-scale SLR systems still encounter big obstacles for realistic applications. To overcome the limitation of current defined Chinese sign gestures, the China Association of the Deaf recently proposed a completely new version of phonology- and radical-coded CSL execution [29]. Each Chinese character can be expressed by executing certain gestures twice using both hands. The first execution of phonology-coded gesture is near the mouth or the chest, representing Mandarin Pinyin. The second execution of radical-coded gesture is near the waist, representing the fore and the end radicals. The related hand shapes and finger orientations are detailed in Figure 2, in which the index (1 to 13) stands for the thirteen basic hand shapes. Each hand shape, with a four-letter name, involves the configuration of a single or multiple finger extension and flexion as shown in Figure 3. Here, we take Mandarin Pinyin “huang” for example. The pronunciation is formed by the fast sounding of basic initial consonant code “h” (Figure 2, Right hand, No.4, horizontal finger orientation) and the basic final consonant code “uang” (Figure 2, Left hand, No. 3, horizontal finger orientation). It is expressed by the two phonology-coded gestures, seen in Figure 4. Common characters “empire”, “yellow”, “lie” share the same pronunciation, but can be distinguished from the fore and the end radicals. These radicals are expressed using two basic radical-coded gestures shown in Figure 5a–c respectively. Consequently, two executions of coded gestures can determine one Chinese character. From Figure 2 we can see that both phonology- and radical-coded gestures share the same 13 hand shapes and only three finger orientation (*i.e.*, with fingers upward, downward and horizontal). Since the number of the coded gestures is limited and constant, and these gestures are relatively simply-designed, the standardization of them become much easier. Therefore, this new CSL version is not only convenient for the deaf people to perform in practice, but also suitable for developing reliable continuous and large-vocabulary-scale CSL recognition systems.

Generally, there are three levels of sign language recognition: finger spelling (alphabets), isolated sign gestures and continuous sign gestures. For finger spelling, the number of hand motions is very small and sometimes mainly finger configuration and orientation information are included [30,31,32]. For isolated sign gestures, usually features that characterize whole hand location and movement as well as appearance features that result from hand shape and orientation are extracted [3,13,33]. When extending isolated gestures to continuous ones, it requires an automatic detection of the boundaries of active gestures so that the recognition algorithm can be applied on the segmented signs [1,21,26,34]. The ultimate goal is to develop a continuous SLR system with a large vocabulary scale. Traditionally, cameras and data gloves are used for data acquisition. They have advantages in capturing gesture information and can obtain a satisfactory classification performance with a relatively large scale of vocabularies. For instance, Fang *et al.* employed Data-gloves as input devices and designed a system that could recognize 5133 isolated Chinese sign gestures with 94.8% accuracy, and distinguish 200 continuous sentences with 91.4% word accuracy [35]. Starner *et al.* used hidden Markov models (HMM) to recognize sentence-level continuous ASL using a single camera. The classification accuracy with a 40-word lexicon was at least 92% [8]. However, when developing continuous SLR systems based on the two kinds of devices, the transition part of two successive gestures, termed as movement epenthesis (ME), should be taken into account. Most studies demonstrate that handling the ME problems yield results superior to those ignoring ME effects [9,36,37]. Gao *et al.* introduced separate HMM to model MEs between each unique pair of signs that occur in sequence [37]. Vogler and Metaxas employed HMM to model MEs between valid combinations of signs [9]. Nevertheless, the training of such systems involves a large amount of data collection, manual data labeling, model training and recognition computation due to the fact that all the MEs between two valid combinations of signs should be considered, leading to heavy training burden [34,38].

Recently, accelerometer (ACC) and surface electromyography (sEMG) sensors are widely adopted for gesture sensing and motion detection [39,40]. ACC can be used to distinguish large scale of movements associated with different trajectories and motions with different initiative and terminative motion orientations. sEMG is capable of capturing rich information of multiple muscular activation and coordination associated with subtle hand, wrist and finger movements. Meanwhile, sEMG has advantages of dealing with movement epenthesis, since the amplitude of sEMG will increase during muscular contraction when performing gestures, but will temporarily keep quiescent baseline during muscular relaxation (*i.e.*, movement epenthesis) [21,41]. Previous studies demonstrated that exploiting the complementary discriminating abilities of both ACC and sEMG sensors can make SLR systems achieve better performance, not only in advancing recognition accuracies, but also in enlarging the number of gesture categories. One example is that Kosmidou *et al.* successfully applied the intrinsic mode sample entropy on combined ACC and EMG data acquired from the dominant hand to recognize 61 isolated Greek sign gestures [16]. Zhang *et al.* proposed a Chinese SLR system with one 3D ACC and five sEMG sensors [40]. They reported a 93.1% word accuracy and 72.5% sentence accuracy for 72 single-handed words forming 40 sentences.

From the linguistic point of view, sign gestures mainly consist of four basic components, summarized as hand shape, orientation, location and movement. Both SCL and international SLs have the same common that some signs share at least one component. Thus, in order to form a large number of signs, only a limited number of patterns of each component are needed. These components are firstly individually classified at the component-level and then integrated together for sign-level classification. This greatly facilitates the development of practically feasible SLR systems. For example, in Liang and Ouhyoung [42], the most number of classes at the component-level was 51 categories (for hand shape), which is smaller than the 71 to 250 sign words that were recognized. Though some work may have small vocabularies (e.g., 22 signs in [9]), their focus is on developing frameworks targeting scalable large vocabularies. Recently, Li *et al.* introduced a subword-level framework for CSL recognition using both ACC and EMG sensors, and the final classification results for 120 subwords and 200 sentences were 96.5% and 86.5% respectively [21]. However, due to the fact that the sign gesture is complex and variant, the movement component is currently modeled for each subword, which indicates that the number of components will still increase when the size of vocabulary in continuous CSL system keeps increasing, leading to huge training burden in practice

In this work, we take the right hand for example and propose a component-level classification framework to validate the feasibility of recognizing phonology- and radical-coded sign gestures using ACC and sEMG sensors. The thirteen basic hand shapes, mainly the subtle extension of single and multiple fingers, will be captured by sEMG sensors. The three finger orientations (U, D, and H) and the two initial positions (mouth or chest, and waist) will be normalized as four different hand movements (*i.e.*, phonology-coded gestures, which are executed upward and horizontal near the mouth or chest will be normalized as moving vertical upward (U) and moving horizontal rightward (R). Meanwhile, radical-coded gestures, which are executed downward and horizontal near the waist will be normalized as moving vertical downward (D) and moving horizontal leftward (L).). These hand movements will be distinguished by ACC signals. To the best of our knowledge, this is the first attempt to design the novel phonology- and radical-coded CSL recognition system using ACC and sEMG signals. Using the newly designed CSL performing strategy, the number of the coded gestures is constant, and the number of components is limited and constant. Besides, all the components are simple palm orientation, hand shapes and movements. Our proposed framework can guarantee the scalability of vocabulary since almost every Chinese character can be made up based on these coded gestures. Besides, it offers an opportunity to reduce the training samples. Although sEMG signals can be unreliable and/or exhibit different characteristics depending on the dynamic nature of motion (*i.e.*, movement artifacts), sweat (The amplitude of sEMG signals will decrease), and level of exertion and fatigue (Median frequency will decline). However, compared with signals of transition movements (*i.e.*, movement epenthesis) and relaxation duration, the amplitude of action sEMG signals can be a burst and quite distinguishable. Recently, a lot of excellent onset detection algorithms, such as Teager Kaiser energy (TKE) operator [43], sample entropy [44], maximum likelihood [45], and EMG Burst Presence Probability (EBPP) [46] can help effectively detect the onset and offset points using sEMG signals under different complicated situations (including above unreliable situations). On the other hand, the proposed CSL are rather simple palm orientation, hand movements and hand shapes. The coded gestures are executed as extending the hand shape along with the trajectory, which gives a lesser chance of the ME problem since the transition movements can be treated as some level of relaxation. Consequently, sEMG signals can provide a feasible scheme for accurate onset and offset detection. Additionally, the number of the components will stay constant even if the number of characters, words or sentences increases. This will ensure the realization of extendable CSL recognition framework with reduced training samples.

The rest of the paper is organized as follows. In Section 2, we will describe the phonology and radical-coded CSL definition and the data acquisition procedure. In Section 3, the related methods will be introduced. Then we will provide the validation experimental results of coded gesture recognition in Section 4, followed by the discussions and conclusions in Section 5.

## 2. Methods

**Figure 6:**
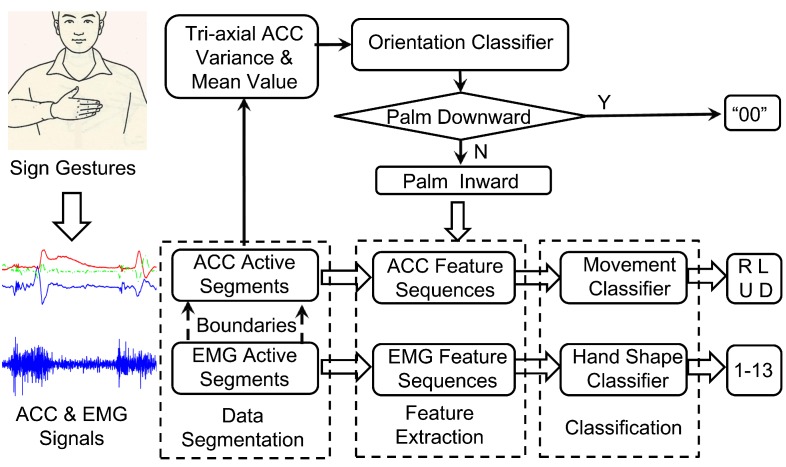
Illustration of the block diagram of the proposed framework.

The entire block diagram of the proposed framework is shown in Figure 6. When subjects execute gestures, multichannel ACC and sEMG signals are synchronously collected. Firstly, data segmentation is performed to determine the onset and offset boundaries of both ACC and EMG active segments. Secondly, the variances and mean values of tri-axial accelerations are calculated for hand orientation recognition. Referring to the sign language gesture definition, only two types of hand orientations, called “Palm Inward” and “Palm Downward” are taken into account. If the “Palm Downward” orientation is detected, it indicates the default sign gesture indexed as “00”. Otherwise, it is the “Palm Inward” orientation gestures. Then both ACC and EMG signal of active segments are divided to frames using the overlapped sliding window techniques, and effective features, with ACC feature sequences for movement recognition and EMG feature sequences for hand shape classification, are sent to corresponding classifiers. The recognized movements are labeled as R, L, U, and D (detailed description in the Section 2.1), whereas the classified hand shapes are marked as 1 to 13. Finally, with the combination recognition results of the hand movement and the hand shape, the coded gesture can be recognized.

### 2.1. Sign Gesture Definition & Data Acquisition

**Figure 7:**
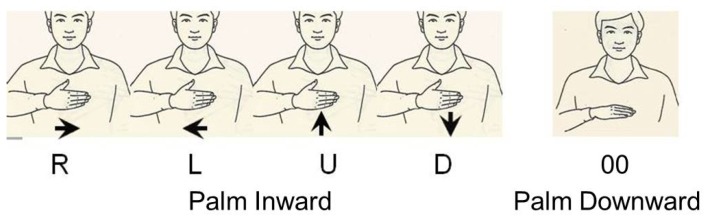
Illustration of the proposed trajectories and the default sign gestures

Without gesture redefinition, the proposed CSL need to classify different static finger orientation (upward, downward and horizontal). Besides, since the hand positions where phonology-code gestures and radical-coded gestures originally executed are different (mouth or chest, and waist), hand position classification is also needed. Both static finger orientation and hand posture recognition based on ACC signals may need stringent normalization, due to individual differences, typically as heights, gesturing habits, and different absolute values related to orientation and position. However, we convert finger orientation and hand position information to four kinds of dynamic hand movements, so that the classification scheme can be much easier and the stringent normalization can be omitted. Take right-handed gestures for example, phonology-coded gestures, with fingers upward and horizontal and near the mouth or the chest, are redefined as holding the hand shape from bottom to top vertically (Vertical Upward, U) and from left to right horizontally (Horizontal Rightward, R). The radical coded gestures, with fingers downward and horizontal near the waist, are redefined as holding the hand shape from top to bottom vertically (Vertical Downward, D), and from right to left horizontally (Horizontal Leftward, L), detailed as Figure 7. The total number of the hand shapes accompanied with different movements, with the “Palm Inward” gesture execution orientation, amounts to 52 (*i.e.*, 13 × 4), which later will be expressed using the index combined with the movement, such as 5R. It means stretching the palm (also referred as five fingers extension) while moving horizontally from left to right. Additionally, a unique “Palm Downward” hand gesture (“00”) is employed on behalf of the actual coded default phenomenon. Totally, 53 gestures are defined. One situation is that the Chinese pronunciation “neng” has unique character “capable”, whose radical-coded gestures will be expressed using “Palm Downward” gesture. Another situation is that some pronunciation contained only the initial consonant, such as character “one”, and only the final consonant, such as character “even”, both of which has one default coded gesture.

**Figure 8:**
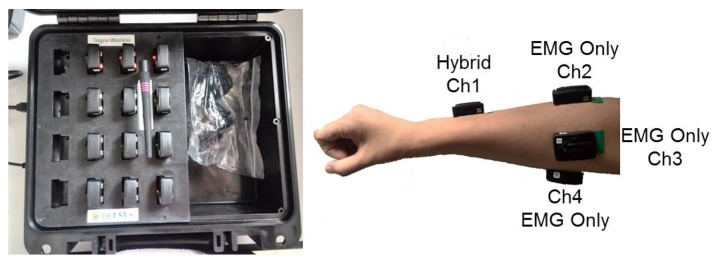
The positions of the ACC and sEMG sensors, as well as the ACC orientations. The “Ch” refers to the channel of EMG sensor.

Similar to the SLR studies in [9,21], the proposed gestures makes full use of component information. However, the advantage of the proposed hand shape components is that they involved neither wrist nor arm movements, but only the subtle finger extension and flexion movements. A number of studies have already demonstrated the feasibility of finger movement recognition using multichannel sEMG signals [41,47,48]. The data acquisition system in this work is Delsys Trigno Lab Wireless System (Delsys Inc. Natick, MA, USA). It has a 16-bit A/D resolution and the sample rates of both EMG and ACC signals are 1927 Hz and 148 Hz. The range of ACC signals is selected as ±1.5 g. The EMG can be configured as EMG only, ACC only and Hybrid mode. Totally one hybrid sensor and three EMG only sensors, which mean one 3D ACC and four sEMG sensors are placed on the skin of muscles of interest (illustrated in Figure 8). The hybrid sensor (labeled as Ch1) aims at the *Muscle (M.) extensor digiti minimi*, *M. extensor pollicis longus* and *M. extensor pollicis brevis* and the other three (labeled as Ch2-Ch4) are placed to target the *M. extensor digitorum*, *M. palmaris longus*, *M. extensor digitorum*, *M. flexor carpi ulnari* and *M. extensor carpi radialis longus*. The sEMG sensor locations are chosen based on optimal scheme, which was proved feasible to reflecting sufficient muscular activation information about finger extension and flexion movements in the previous studies [40,47].

### 2.2. Data Segmentation

**Figure 9:**
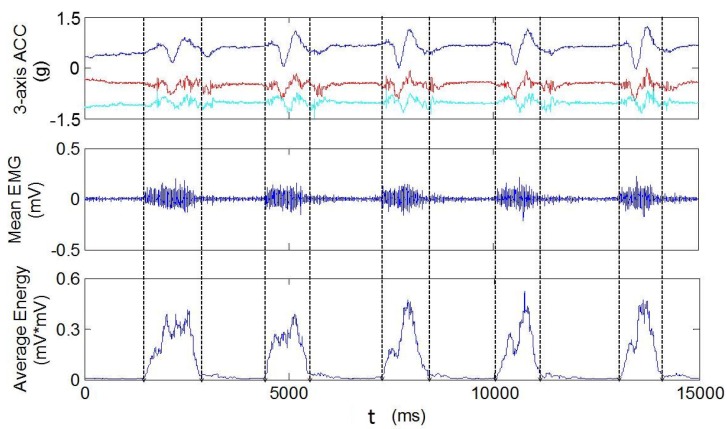
An example of the segmentation results of sequential right-handed gestures.

The intelligent processing of sign language gesture recognition needs to automatically determine the onset and offset boundaries of active signal segments. Previous studies demonstrate that sEMG signals have natural advantages in mitigating or avoiding the movement epenthesis problem, owning to the fact that the EMG signals are considered as instantaneous indicators of muscular activation associated with the implementation of sign gestures. Thus, several characteristics of the EMG signals can be used to perform data segmentation, e.g., the average energy, the envelope, and the root mean square (RMS) [43,49]. In this work, the overlapped sliding averaged energy is adopted for data segmentation (called Sliding Average in the following). The size of the sliding window is 64 points, and the overlap is 1 (which meant sliding one point by one point). An example of the segmentation results of sequential right-handed gestures is illustrated in Figure 9. The signals of the tri-axial ACC and the averages of the 4-channel EMG signals (Ch1-Ch4) are shown in the first and second rows respectively. The segmentation results based on sliding average are provided in the last row. It is obvious that all the active segments have been correctly detected.

One issue of sliding average is to determine the onset and off thresholds. Previous studies [21,40] suggested that the level of muscle activity for a signer with respect to the background noise should be taken into account. Thus both the optimal thresholds are the same, determined as several percent of the corresponding mean average energy value when the hand grasp task was performed at the signer’s maximal voluntary contraction (MAV) on the basis of the mean average energy corresponding to background noise. Based on experimental results, 2% is quite suitable.

### 2.3. Component-Level Classification

The sign components employed in this work include palm orientations, hand movements and hand shapes. The feature sets describing each component are extracted and taken as the inputs of corresponding component-level classifiers respectively. In order to distinguish two palm orientations, both the mean and variance values of ACC signals of the active segments are extracted. Then, to represent four different hand movements, time-normalized 32-points ACC sequences are extracted for each active segment. Finally, the mean absolute vales (MAV) and multi-order (usually 4 is optimal) autoregressive model coefficients (ARs) are extracted to represent different hand shapes.

#### 2.3.1. Hand Orientation Classifier

Orientation refers to the direction towards which the hand is pointing or the palm is facing. Generally, the inclination information with respect to the reference planes interpreted from ACC can be used to estimate the orientations. In this work, only two types of orientations are included, Palm Inward and Palm Downward. The palm inward gestures always keep parallel to the body and associated with trajectories, whereas palm downward ones keep perpendicular to the body and static. The mean variance of resultant ACC (root mean square of the three-axial ACCs) and mean value of each ACC axis for each active segment, totally 4 dimensional feature vectors, are extracted to represent the two orientations. The popular linear discriminant classifier (LDC) [50], based on the Bayesian criterion, is trained to model the probability density as a Gaussian distribution:

(1) $$P\left(\theta |\Theta_{i}\right)=G\left(\theta ,\mu_{i},\Sigma_{i}\right)$$

where $\mu_{i}$ and $\Sigma_{i}$ are the mean vector and covariance matrix of multivariate Gaussian distribution $G(\cdot ,\mu_{i},\Sigma_{i})$ of the *ith* hand orientation class. $\Theta_{i}$, $\mu_{i}$ and $\Sigma_{i}$ can be estimated by training samples of hand orientation class $\Theta_{i}$.

#### 2.3.2. Hand Movement Classifier

Hand movements of SL involve changes of orientations and trajectories related to hand and forearm motions. In this work, the hand movements are simplified into four directional trajectories, generally known as Upward, Downward, Rightward and Leftward, and are marked with the first capital letter (U, D, R and L). Many algorithms used for ACC-based trajectory recognition, are either evaluating the similarity matching degree of time series, or recognizing patterns represented by feature vectors extracted from time series, typically known as correlation functions, K-mean clustering algorithms and K nearest neighbor (KNN), *etc*. One big challenge of SRL system is the different speeds between the sample gestures executed by different people and even the same person in different times. The effect can be weakened by time scale normalization. However, when it comes to local changing speed, the similarity degree may decrease fast. Dynamic time warping (DTW) algorithm is employed in this work. It has the ability of similarity measurement between the two sequences varied in time or speed, even during the course including accelerations and decelerations [51,52,53].

In order to build hand movement classifier, for each active gesture segment, ACC signals are normalized to fixed-point time sequences. Training and testing sequences are represented as $T_{i}=\left\{t_{1},t_{2},...,t_{m},...,t_{N}\right\},i=1,2,3$ and $R_{j}=\left\{r_{1},r_{2},...,r_{n},...,r_{N}\right\},j=1,2,3$ where *N* is the total number of feature vector dimensions. For each axis, the distortion degree of $d_{i}\left(t_{m},r_{n}\right)$ is defined as the Euclidean distance (Equation (2)). For fast computation, some conditions are added to restrain the searching path, with the path slope smaller than 2 and larger than 1/2. Thereby, the cost function $D_{i}\left(t_{m},r_{n}\right)$, meaning the accumulative distortion value from the starting point $\left(t_{1},r_{1}\right)$ to $\left(t_{m},r_{n}\right)$ is calculated as Equation (3). When it comes to the end point $\left(t_{M},r_{N}\right)$, the whole DTW cost of each axis can be obtained. The overall DTW cost is defined as Equation (4).

(2) $$d_{i}\left(t_{m},r_{n}\right)=\sqrt{{\left(t_{m}-r_{n}\right)}^{2}},i=1,2,3$$

(3) $$D_{i}\left(t_{m},r_{n}\right)=d_{i}\left(t_{m},r_{n}\right)+\min\left(D_{i}\left(t_{m-1},r_{n}\right),D_{i}\left(t_{m-1},r_{n-1}\right),D_{i}\left(t_{m},r_{n-1}\right)\right),i=1,2,3$$

(4) $$D\left(t_{M},r_{N}\right)=\sum_{i=1}^{3}D_{i}^{2}\left(t_{M},r_{N}\right)$$

In this work, DTWs between the unknown testing sample and all the training samples are computed. Then, an averaged DTW can be derived from all the DTWs of one trajectory. Consequently, four averaged DTWs can be obtained. The unknown testing sample will be classified into the movement category, which achieved the smallest averaged DTW.

**Figure 10:**
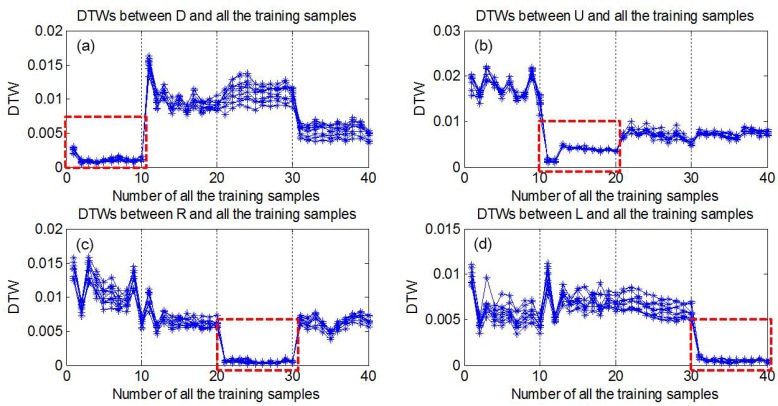
Illustration of DTW cost between partial testing samples and training center. (**a**) Testing samples of movement D; (**b**) Testing samples of movement U; (**c**) Testing samples of movement R; (**d**) Testing samples of movement L.

Figure 10 illustrates all the DTWs between testing samples and all the training samples. From Figure 10a–d, the testing samples correspond to trajectory D, U, R and L, respectively. For each trajectory, both 10 testing samples and 10 training samples are illustrated. Besides, the training samples sequentially correspond to trajectory D, U, R and L. From Figure 10a, it can be seen that, for testing samples corresponding to trajectory D, the smallest DTWs are derived from the first 10 training samples (seen in red dashed rectangle) corresponding to the same trajectory. It indicates that the averaged DTW of all the training samples of trajectory D is the smallest. For other trajectories, the same conclusion can also be drawn. It displays that the proposed DTW algorithm can represent the similarity of two sequences and can be employed for hand movement classification.

#### 2.3.3. Hand Shape Classifier

The hand shapes, referred to configuration of finger movement, from a single finger to all five fingers extension or flexion, are associated with muscular activity reflected by sEMG signals. Various sEMG features are previously studied for myoelectric patterns recognition [54,55,56]. Mean absolute value (MAV) and autoregressive model coefficients (ARs) demonstrate outstanding classification performance in gesture recognition with low computational burden, and they show superiority in real-time SLR systems [21,57]. Thus, for each sEMG channel, both MAV and 4-order ARs are extracted.

HMM is a stochastic process that takes time series as the input and the output is the probability calculated by the model trained using the input time series data [58]. It includes double stochastic process composed of Markov chains and general stochastic process. The formal definition of a HMM is as follows: λ=(A,B,π,N,M), where *N* is the number of hidden states, *M* is the number of possible observations of each state, *A* is the state transition probability matrix, *B* is the observation probability distribution given the state, and *π* is the initial state probability distribution, usually uniform at the beginning. Lee proposed a HMM framework for an automatic speech recognition scheme using solely EMG information [59]. Chan and Englehart proposed a HMM framework for recognizing six classes of limb movements using four channel EMG signals. Both of them achieved high classification accuracies [60]. In this work, we also adopted HMM for hand shape recognition. First, overlapped sliding windows are employed to divide the active EMG segment into several frames. The size of the sliding window is 128 points, and the overlap is 64 points. Then the EMG feature vectors are extracted frame by frame, forming the observation sequences, marked as $O_{R}$. Afterwards, $O_{R}$ is utilized to train HMM using Baum-Welch algorithm [61] and denoted as $\lambda_{c}$, where *c* is the index of the hand shapes to be recognized. The structure of HMM is left-to-right with 5 hidden states and the number of Gaussian mixture variable is 3 [21,40].

For an unknown hand shape, the likelihood $P(O\left|\lambda_{c}\right.)$ through each candidate *c* can be calculated by forward-backward algorithm, where 1≤c≤C. *C* is the total number of hand shape categories. Then the unknown hand shape will be recognized as class $c^{*}$, whose HMM achieves the maximum likelihood:

(5) $$c^{*}=\underset{c}{\arg\max}\left(P\left(O\left|\lambda_{c}\right.\right)\right)$$

## 3. Experiments

### Data Set Construction

With the approval of the Ethics Review Committee of Hefei University of Technology, five healthy and right-handed volunteers (two females and three males), aged between 25 and 28 years old, were recruited for this study. All the volunteers were well-trained and familiar with the CSL and they were also the volunteers in a local school for the hearing impaired. During the experiments, each subject was asked to naturally and continuously perform predefined gestures one by one at a moderate speed. Each gesture was instructed to be performed by extending the hand shape along with the movement. When a gesture was finished, there followed a brief and natural relax for about 1–2 s.

**Figure 11:**
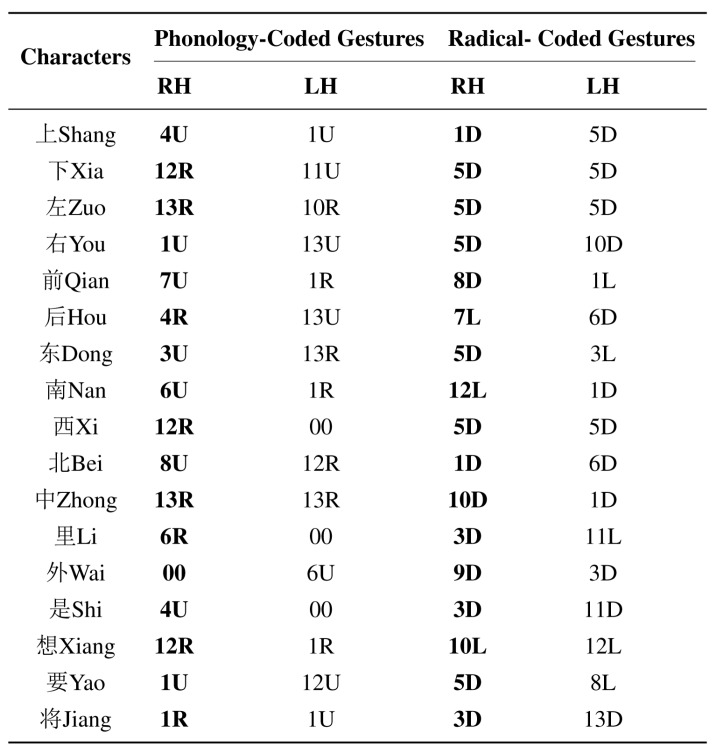
Typical Chinese characters with corresponding coded gestures.

In total, 223 frequently used characters were selected, with each having four coded gestures (two left-handed gestures and two right-handed gestures). Figure 11 illustrates some typical Chinese characters and their corresponding coded gestures. In this work, without loss of generality, only the right-handed gestures were recognized. The data collection procedure was as follows. First, the 53 isolated coded gestures were sequentially executed with each having 20 repetitions during one experimental duration. Totally five experimental durations were finished across five different times. Then, all the right-handed coded gestures of 223 characters were executed with each having one repetition during the sixth duration. During the coded gesture recognition task, both training samples and testing samples came from isolated coded gestures. Meanwhile, during the continuous Chinese character recognition task, isolated coded gestures were used for training, and coded gestures derived from characters were used for testing. By the way, Chinese character recognition task employed 9 repetitions of each hand shape within each hand movement for training (totally 36 samples for each hand shape).

## 4. Results

### 4.1. CSL Coded Gesture Classification Results

In this part, isolated coded gestures from training set are first employed to evaluate the feasibility of the proposed component-level algorithm. According to our proposed gesture definition in this study, hand movements are not sensitive to the changing of hand shapes. On the one hand, hand movements are represented only by ACC signals, whereas the hand shapes are represented only by sEMG signals. By this means, The ACC signals are relatively independent on sEMG signals. On the other hand, the proposed gesture definition of hold the hand shape during one certain hand movement promises that hand movements are not sensitive to the changing of hand shapes. Thus, training samples from different coded gestures sharing the same hand movement can be used to train hand movement classifier, whereas those from different coded gestures sharing the same hand shape can be used to train each HMM model. It is noted that all the coded gesture classification tasks are performed in a user-specific manner. Both the testing data and training data were from the same volunteer.

#### 4.1.1. Palm Orientation Classification Results

Regarding the palm orientation classification task, only two kinds of orientations were involved (*i.e.*, Palm Inward and Palm Downward). The proposed features including variance and mean values extracted from the ACC signals, as well as the LDC classifier are quite effective to distinguish the orientations. The experimental results demonstrate that all the palm orientations are totally correctly classified.

#### 4.1.2. Hand Movement Classification Results

**Figure 12:**
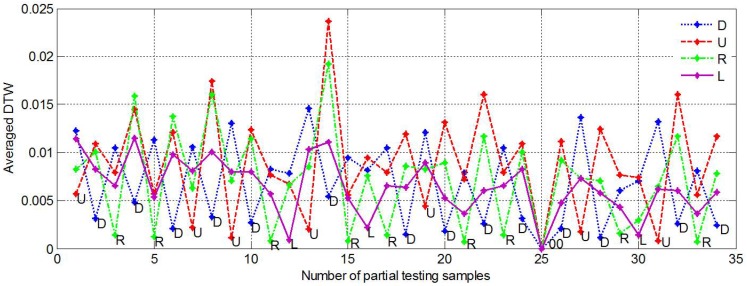
The movement classification results of several Chinese characters based on DTW.

After the palm orientation classification, only palm inward gestures were further processed. First, the proposed DTW algorithm was employed for similarity distance calculation, and then the averaged DTWs were calculated. Figure 12 demonstrates the recognition results of hand movements. Different colors of stars represented different types of movements. For each testing sample, the smallest averaged DTW was marked with its type and it was also the identification result. The testing samples were selected as shown in Figure 11 (Bolded, Right-handed only) and the movements were sequentially (U D), (R D), (R D), (U D) *etc*. row by row. It was found that all the movements were correctly recognized. Previous study has demonstrated that using DTW algorithm, the averaged recognition accuracy of 8 types of gesture patterns could reach to 98.6% [62]. In this work, the averaged DTW helped the recognition accuracy of 4 types of hand movements to achieve 100%, which also attributed to the obvious differences between the four movements. Additionally, the classification of hand movements did not depend on hand shapes. In other words, no matter which type of hand shape is executed, such as 1U, 11U or 13U, the movement will be classified as U. It indicated from Figure 10 that about 10 repetitions of training samples for each movement were enough to achieve satisfactory performance and the number of training samples used for hand movement classification is not burdensome.

#### 4.1.3. Hand Shape Classification Results

**Figure 13:**
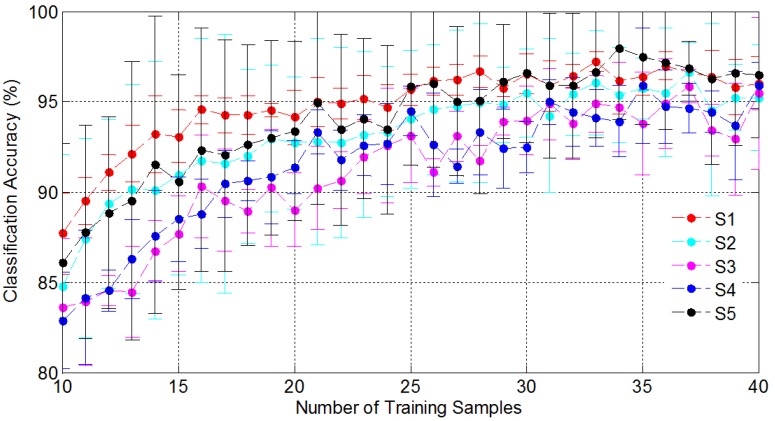
The overall classification results of hand shapes with different numbers of training samples for all the subjects.

HMM was adopted for hand shape recognition. First, the coded gestures were employed to evaluate the classifier performance. Figure 13 presents the overall classification results of hand shapes with different training numbers for all the volunteers. S1 to S5 represent the five volunteers. For each selected training number, the training samples were randomly selected for six times for each subject. Thereby, the averaged classification accuracies as well as the standard deviation are shown in Figure 13, which demonstrates that increasing training samples leads to the growth of recognition rates. For instance, when the number of training samples was 10, the overall classification was the least, about 85%. However, when the number surpassed 30, most of the overall classification accuracies kept satisfactory and stable (above 94%, at least 92%). The results indicate that the 13 hand shapes can be effectively distinguished by HMM while only about 30 repetitions of each hand shape for each signer needed to be implemented. It proved that the sEMG signals were rather consistent when the same hand shape was performed along with different hand movements. However, to keep the homogeneity of training samples, it is suggested that 8 or 9 repetitions of each hand shape within each movement (in total four movements) should be implemented. Such training burden is acceptable in practice.

### 4.2. Continuous Chinese Character Classification Results

After evaluating the classification performance of aforementioned components, it can be seen that it is feasible for the proposed method to perform Chinese character classification based on phonology- and radical-coded gestures. The aforementioned data segmentation algorithm was first employed to automatically detect the meaningful active gesture segments within continuous execution of testing set, and the segments were then to be identified and labeled as sequences by recognized coded gestures. Without semantic information intervention, the continuous Chinese character recognition was mainly manifested by the coded gesture classification. Table 1 illustrated the continuous classification results of both coded gestures and characters. Each character consisted of two right-handed coded gestures, and the total coded gesture number of all the 223 characters is 446. It was found that the classification accuracy of continuous coded gestures was at least 94.84%, and it could reach above 97%. Besides, overall classification results of continuous characters varied from 90.58% to 94.17%. Due to the fact that one character has two coded gestures, and the misclassified coded gestures might come from the same character, the actual number of misclassified characters was smaller than that of misclassified gestures, such as S2. Meanwhile, compared to the isolated coded gesture classification results with the same number of training samples (varying from 94.73% to 97.68%), the continuous recognition accuracies of coded gestures were only slightly decreased, which indicated that the proposed gesture performing style would not be seriously impacted even though the same coded gesture appeared in different Chinese character expression. It indicates that the number of training samples will not be intensely increased when vocabulary scale increases and it can still maintain an acceptable level.

**Table 1:** The overall classification accuracies of continuous characters.

Subjects	Real Number		Identified Number		Accuracies (%)	
	Gestures	Characters	Gestures	Characters	Gestures	Characters
S1	446	223	433	210	97.09	94.17
S2	446	223	430	208	96.41	93.72
S3	446	223	428	205	95.96	91.93
S4	446	223	427	204	95.74	93.27
S5	446	223	423	202	94.84	90.58
Overall	2230	1115	2141	1029	96.01	92.73

## 5. Discussions and Conclusions

So far, we have demonstrated the feasibility of CSL recognition based on phonology- and radical-coded gestures using the combination of ACC and sEMG sensors. It is our first attempt to develop a systematic framework to represent Chinese characters in the form of text-type sign language. This coded strategy takes full account of the unique properties of Chinese characters, and provides the probability of representing the Chinese character by the fixed number of coded gestures. This will ensure the realization of extendable CSL recognition framework with reduced training samples. The superiority of the combined utilization of EMG and ACC sensors in the realization of sign language recognition system has been noted by several previous studies. Table 2 summarizes several representative relevant work and our proposed. The proposed work demonstrates a significant progress on designing continuous and scalable SLR systems, considering the small number of basic units (coded gestures), the considerable characters scale and the achieved satisfactory character recognition rates. Some studies have demonstrated that basic units-based approach achieved exciting recognition accuracies and can contribute to a continuous CSL recognition system with a larger vocabulary scale [20,21,35]. However, the work studied by C. Wang [20] ignored the homonyms phenomenon of Chinese characters. Thus in this paper, we added radical encoding information to help distinguish different homophones. And we made a great progress on reducing the number of training samples compared with Li’s work [21]. In Li’s work, they summarized a vocabulary of 120 sub-words to express the 181 frequently used CSL words, forming the defined 200 sentences. For each data collection procedure, the former two repetitions of each sub-word were employed for training, and the third repetition was for testing. It was obvious that the number of training samples was large and it would increase with the scale of sub-word increased. However, the proposed completely new version of phonology- and radical-coded CSL execution scheme has limited and constant number of basic units, and the training samples will not intensively increased as the number of characters increases. Additionally, EMG-based coded gesture recognition technique has natural advantages of dealing with movement epenthesis, and ensures a satisfactory classification accuracy.

The current data set was just a typical assessment example to explore the feasibility of the proposed CSL recognition framework. To evaluate the proposed framework for quite a large vocabulary and smoothing communication situation, a lot of efforts still need to be made. One limitation of current study is that decision-level integration algorithms are needed. If one coded gesture is misclassified, the character will be misclassified. Due to the fact that phonology basic units obey the rules of the initials and finals, if one certain phonology coded gesture is performed, the following possible radical coded gestures are determinable and kind of limited. Context information about phonology and radical rules can be utilized for character-level fusion classification. Secondly, only right-handed coded gestures are currently considered since the right hand is the dominating one for most people. Studies demonstrated that sEMG signals from both hands of the same person are different. The classification performance of left-handed gestures should be further studied. Another issue to be considered is that currently the proposed CSL recognition framework focuses much on the evaluating the feasibility of recognizing coded gestures in a signer-dependency style, the performance of signer-independency classification should be further investigated. Besides, the acceptability of this kind of gesture performing style, as well as the time spent for learning among school-age students should be further investigated.

**Table 2:** List of some related work.

Author	Sensor Types	Language	Isolated/	Basic Units	Vocabulary	Accuracy (%)
	per Hand		Continuous			
Fang [37]	1 CyberGloves	Chinese SL	Continuous	Phonemes	5113 Phonemes	91.9
	3D tracker					
Losmidou [16]	1 3-axis ACC	Greek SL	Isolated	Word	60 words	>93
	5 bipolar EMG					
Zhang [40]	1 3-axis ACC	Chinese SL	Continuous	Word	72 words	>95
	5 bipolar EMG				40 sentences	72.5
Li [21]	1 3-axis ACC	Chinese SL	Continuous	Component	121 subwords	96.5
	4 bipolar EMG				200 sentences	86.7
The proposed	1 3-axis ACC	Chinese SL	Continuous	Phonology	53 basic units	>95
	4 bipolar EMG			& Component	223 characters	>92
